# Delayed completion of total gastrectomy for refractory fistula following laparoscopic sleeve gastrectomy: A case report and literature review

**DOI:** 10.1097/MD.0000000000043748

**Published:** 2025-08-15

**Authors:** Gerelt-Od Khenmedekh, Dae Hoon Kim

**Affiliations:** a Department of Surgery, Chungbuk National University College of Medicine, Cheongju-si, Republic of Korea; b Department of Surgery, Chungbuk National University Hospital, Cheongju-si, Republic of Korea.

**Keywords:** bariatric surgery, case report, completion total gastrectomy, laparoscopic sleeve gastrectomy, refractory fistula, stapler line leakage

## Abstract

**Rationale:**

Laparoscopic sleeve gastrectomy (LSG) is a widely utilized and effective procedure in metabolic and bariatric surgery (MBS). Stapler line leakage is one of the most catastrophic complications of LSG. Although there are various available treatment options for refractory leakage, there is currently no standardized protocol. In this case report, we present a treatment option for a chronic gastric fistula following LSG, performed as a salvage treatment after the failure of several conservative management techniques. We also reviewed the literature to contextualize our case within the spectrum of treatment strategies for refractory fistulas.

**Patient concerns:**

A 45-year-old woman underwent LSG 7 days prior to presentation. The patient presented to the emergency department with abdominal pain and fever.

**Diagnoses:**

She was diagnosed with a gastric leak following LSG.

**Interventions:**

The patient underwent an emergency surgery for generalized peritonitis and hemodynamic instability, during which multiple drains were placed in the abdomen. Furthermore, the patient was managed conservatively, including endoscopic vacuum therapy for 3 months.

**Outcomes:**

The leakage persisted despite these interventions. Complete total gastrectomy was performed 3 months later, and the patient was discharged 27 days after the surgery.

**Lessons:**

If conservative treatments fail to resolve leakage, delayed total gastrectomy should be considered as a definitive treatment option for refractory leaks.

## 1. Introduction

Laparoscopic sleeve gastrectomy (LSG) is one of the most frequently performed metabolic and bariatric surgeries (MBS) globally. It involves removal of approximately 70% to 80% of the greater curvature of stomach, significantly reducing the gastric volume while preserving gastrointestinal continuity.^[1,2]^ Staple line leakage (SLL), one of the most serious complications of LSG, has been reported in 0.5% to 7% of cases.^[1–3]^ Leakage is the second most common cause of mortality, with reported rates ranging from 0.1% to 3.7% of all MBS.^[4–6]^ SLL treatment begins with conservative management and is followed by aggressive surgical intervention. Leaks which do not respond to conservative treatment and last longer than 12 weeks, categorized as a chronic leakage or fistula, such leaks develop in approximately 7% of cases.^[7–9]^ Chronic leaks are challenging to treat because of their persistence. If all conservative approaches fail after 3 months, surgical reconstructive procedures may be required. We report a successful delayed total gastrectomy as salvage treatment for a patient with a persistent, refractory fistula unresponsive to 3 months of conservative management after LSG. This case highlights the importance of considering surgical intervention, such as total gastrectomy, as a definitive solution in complex and refractory cases of SLL.

## 2. Case presentation

**Figure 1.**
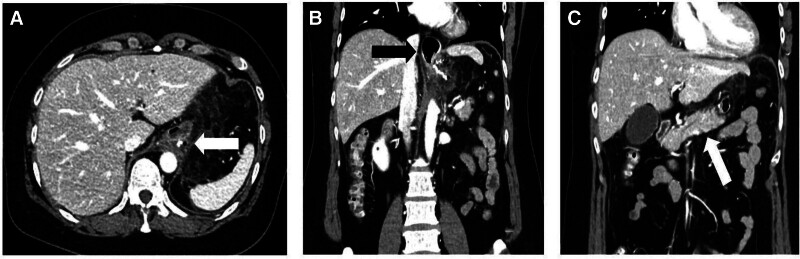
Abdominal CT performed during the initial presentation to the emergency department. (A) The white arrow indicates an air bubble with intra-abdominal fluid collection. (B) The black arrow indicates the dilatation of the upper gastric portion. (C) The white arrow indicates peritoneal wall enhancement. CT = computed tomography.

A 45-year-old (body mass index, BMI: 30.1 kg/m^2^) woman presented to the emergency department with complaints of fever and abdominal pain. The patient had undergone LSG at a local hospital 8 days prior. In the emergency department, a computed tomography (CT) was performed, revealing air bubbles with fat infiltration (Fig. 1A) and dilatation of the proximal part of the sleeve-shaped stomach (Fig. 1B), with no evidence of dilatation in the distal part of the sleeve-shaped stomach (Fig. 1C). However, this finding was overlooked, and the patient was discharged. Two days later, she returned to the emergency department in shock with hemodynamic instability. Her vital signs were as follows: BP, 68/30 mm Hg; pulse rate, 126 bpm; and respiratory rate, 20 breaths/min. The patient had a medical history of hypertension but denied any relevant family history. Physical examination revealed generalized rebound tenderness and abdominal distention.

Laboratory investigations revealed significant metabolic acidosis, azotemia, and hyponatremia. Key findings included a pH of 7.19, pO_2_ of 146 mm Hg, pCO_2_ of 32.8 mm Hg, bicarbonate (cHCO_3_‐) of 12.2 mEq/L, and lactic acid of 1.2 mmol/L. Renal function tests showed a creatinine level of 5.86 mg/dL, an estimated glomerular filtration rate of 8.0 mL/min/1.73 m², and blood urea nitrogen of 98.1 mg/dL. Additionally, the serum sodium level was markedly low at 124 mmol/L, and the D-dimer level was elevated at 7.2 mg/L.

**Figure 2.**
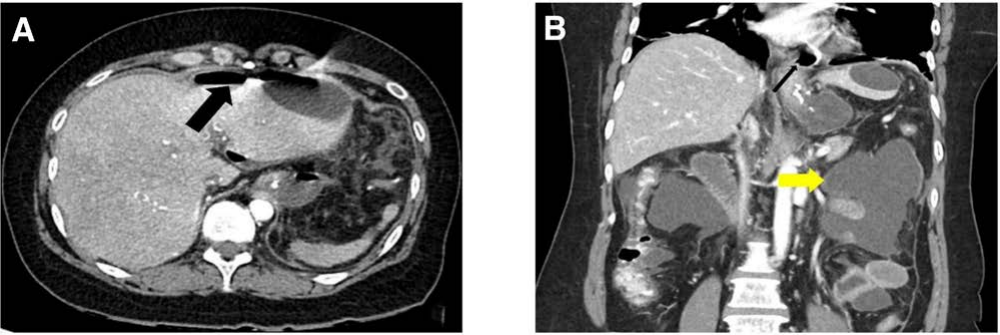
The second abdominal computed tomography scan. (A) The black arrow indicates the presence of free air. (B) The yellow arrow indicates an excessive intra-abdominal fluid collection, with associated wall enhancement, as observed in the frontal view.

During the second admission, abdominal CT scanning revealed the presence of free air (black arrow, Fig. 2A). Additionally, excessive intra-abdominal fluid collection (yellow arrow) and peritoneal wall enhancement were observed in the frontal view on the abdominal CT (Fig. 2B). Based on the patient’s overall condition, imaging studies, and diagnostic laparoscopy, the patient was diagnosed with stapler line leakage following LSG, abdominal adhesiolysis, and peritonitis.

**Figure 3.**
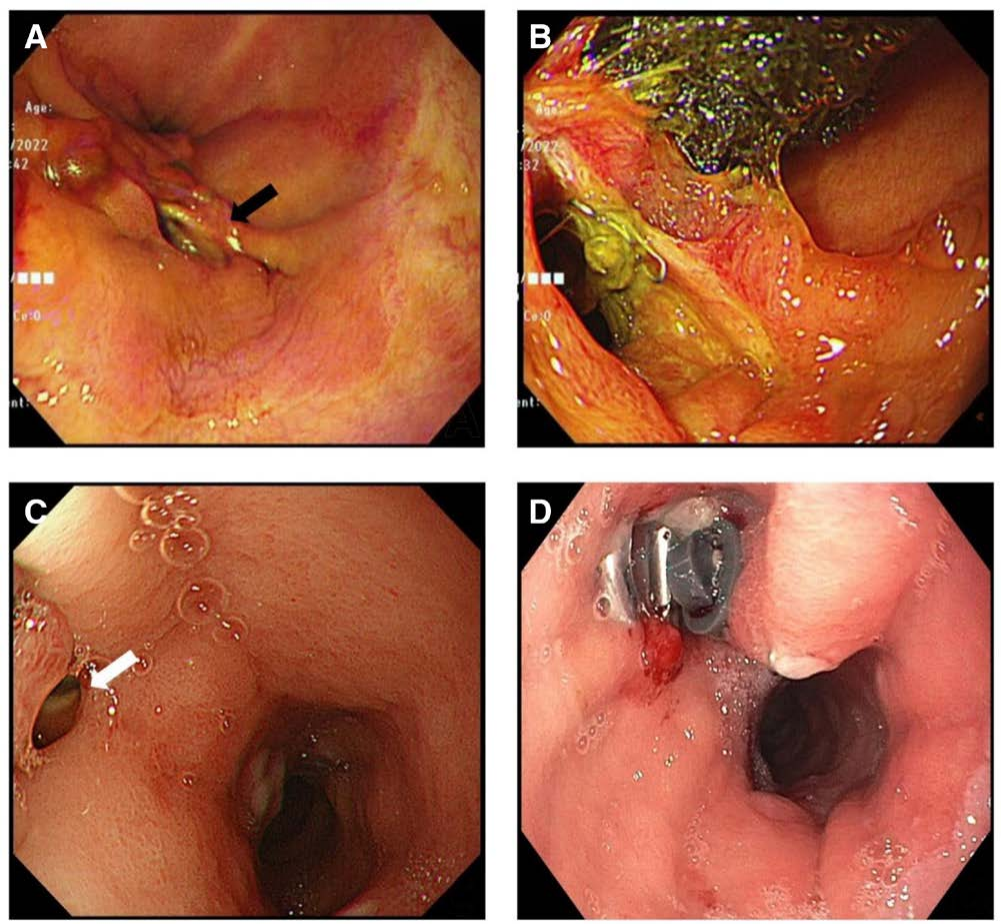
Images from ESD revealing different periods and types of conservative treatment. (A) EGD was performed the day after the initial procedure revealed an orifice of the perforation (black arrow) located at the end of the stapler line. (B) EVT was initiated adjacent to the perforation. (C) Two months after the initial EVT procedure, no signs of healing were observed (white arrow). (D) On 86th days after the initial EVT, a conventional clip was applied; however it was also unsuccessful. ESD = esophagogastroduodenoscopy, EVT = endoscopic vacuum therapy.

Firstly, we performed exploratory laparoscopy. Intraoperative findings revealed that the perforation site could not be clearly identified due to severe abdominal inflammation, bowel edema, and extensive intra-abdominal adhesions. Therefore, the surgical approach focused on adhesiolysis, multiple drain placement, and extensive irrigation. Jackson-Pratt drains were placed near the suspected leakage site in the right lower quadrant. On the day after the initial procedure, the patient underwent esophagogastroduodenoscopy (EGD), which revealed an orifice of perforation (black arrow) at the top end of the staple line, approximately 1 cm from the esophagogastric junction (Fig. 3A). Purulent discharge was observed and suctioned during the procedure. Endoscopic vacuum therapy (EVT) was initiated as part of the initial conservative management, with and EVT sponge placed adjacent to the perforation site (Fig. 3B). The EVT sponge was replaced twice weekly. Additional conservative treatments were administered, including nutritional support, antibiotic therapy, proton pump inhibitors, and percutaneous catheter drainage. Empiric antibiotic therapy was initiated with a combination of ceftriaxone and metronidazole while awaiting culture and sensitivity results. Once these results were available, the antibiotic regimen was adjusted based on the sensitivity profiles.

Despite these interventions, the patient’s clinical condition did not improve significantly. Two months after the initial EVT, EGD revealed no evidence of healing at the leakage site (white arrow, Fig. 3C). The patient continued to exhibit signs of peritonitis, and abdominal CT confirmed persistent intra-abdominal fluid collection associated with peritonitis. Repeated EGD evaluations demonstrated an ongoing failure of the leakage to heal.

Over the following weeks, the patient’s condition progressively deteriorated; and was complicated by stress-induced cardiomyopathy, pneumonia, pleural effusion, cardiorenal syndrome, and ultimately, multiple organ dysfunction syndrome. Despite aggressive conservative management and therapeutic interventions to address the multiple organ dysfunction syndrome, surgical treatment was deferred in favor of continuing EVT to manage the leakage. Regular monitoring with EGD showed no significant progress in the healing process.

At 86 days after the initial EVT, advanced endoscopic measures were performed to treat the refractory fistula. These include the application of conventional clips, band ligation, and over-the-scope clips (OTSC) (Fig. 3D). Unfortunately, these interventions failed to achieve leakage closure. Despite the comprehensive use of EVT and other conservative treatments, no signs of healing were observed. Therefore, we decided to perform total gastrectomy (TG) with Roux-en-Y esophagojejunostomy and feeding jejunostomy 94 days after the initial procedure.

**Figure 4.**
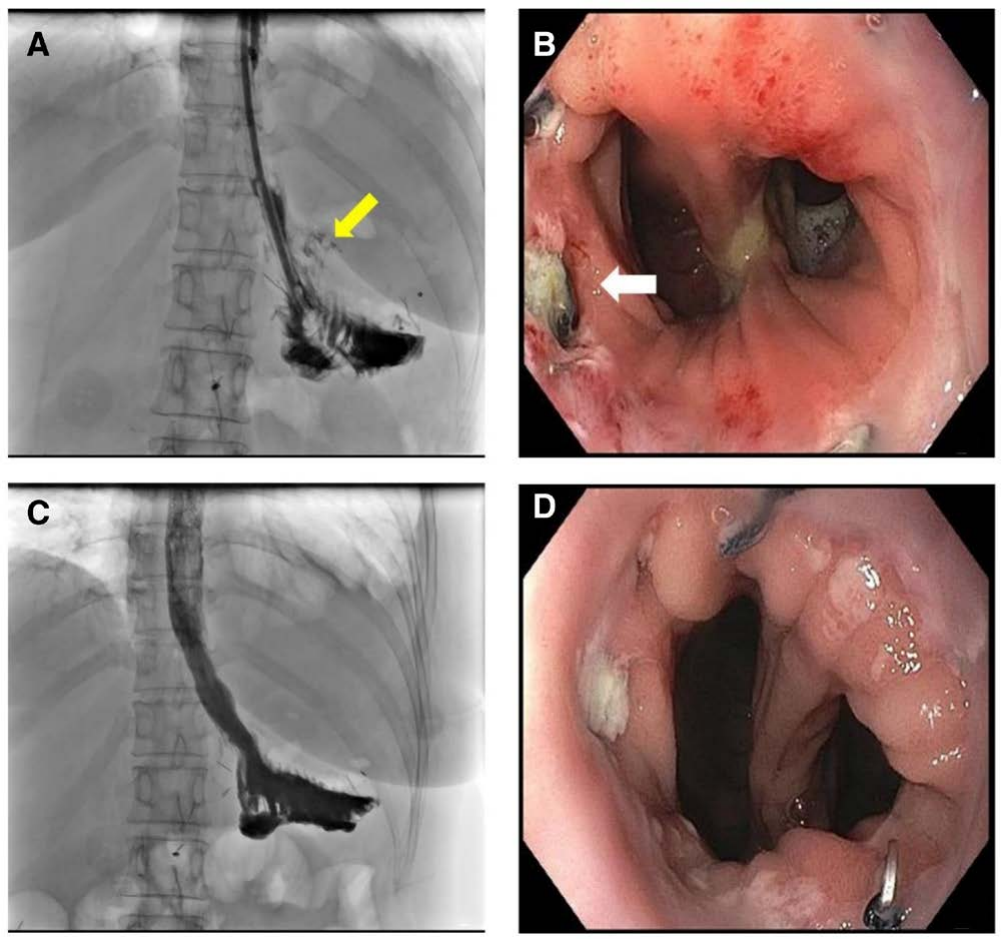
Images from follow-up after total gastrectomy (TG). (A) A leak at the esophagojejunostomy site after TG was identified on an UGI study with barium (yellow arrow). (B) The leakage was confirmed via esophagogastroduodenoscopy (EGD) (white arrow). (C and D) shows that by the 10th day of EVT, the esophagojejunostomy leakage had successfully healed, as confirmed by both UGI and EGD. EGD = esophagogastroduodenoscopy, EVT = endoscopic vacuum therapy, UGI = upper gastrointestinal study.

After performing TG, an upper gastrointestinal study with gastrografin and EGD was conducted to assess postoperative complications. However, a leakage at the esophagojejunostomy site was noted (Fig. 4A and B). We performed EVT to manage the leakage, which facilitated healing for over 10 days during the patient’s inpatient care. The patient was discharged in a stable condition 27 days after TG, and the esophagojejunostomy leakage healed successfully (Fig. 4C and D). The patient’s BMI was monitored throughout the treatment period and during follow-up. During the treatment duration, the BMI fluctuated between 24.41 kg/m^2^ and 27.18 kg/m^2^. At the time of hospital discharge, the patient’s BMI was measured 24.47 kg/m^2^. Notably, at the most recent follow-up examination, the patient’s BMI was decreased to 20.42 kg/m^2^.

## 3. Discussion

LSG, one of the most popular procedures in MBS, is increasingly favored because of its effectiveness in treating severe obesity and improving metabolic diseases, including diabetes. Some studies have reported that LSG is associated with a lower complication rate compared with other types of bariatric surgery.^[1–3]^ Approximately 75% to 90% of these leaks occur at the proximal third of the staple line along the greater curvature, a region that is particularly challenging to manage. This segment of the stomach is more susceptible to reduced blood flow, has a thinner gastric wall, and increased intraluminal pressure, often exacerbated by distal stenosis or twisting at the gastric angle. Additionally, this area is generally weaker than other regions in the absence of digestive suture.^[1,10–12]^

Jain et al^[13]^ previously reported that staple line leaks typically occur within 2 distinct timeframes: within the first 48 to 72 hours postoperatively due to technical failures, or after 5 to 7 days, often resulting from ischemia. Ischemic leaks are attributed to the complex vascular anatomy of the stomach and can lead to compromised blood flow and subsequent staple line failure. In contrast, mechanical leaks occur earlier and are often associated with immediate intraoperative factors.

Prior studies have identified several risk factors for gastrointestinal leakage, which can be categorized as either patient-related, surgery-related, and surgeon-related.^[12–17]^ In obese patients, high BMI is considered a potential risk factor, which possibly acts by increasing tissue complexity, which can present challenges during stapling, thereby increasing the likelihood of leakage following sleeve gastrectomy.^[12,16]^ Furthermore, both preoperative and intraoperative hypotension have been associated with an elevated risk of leakage, potentially due to inadequate perfusion of the surgical site. Simon et al^[15]^ previously reported that episodes of systolic blood pressure lower that 100 mm Hg lasting 15 minutes and 20 minutes were significantly linked to staple line leakage, with *P*-values of .027 and .008, respectively. In addition, intraoperative factors such as bougie size and the technique used for gastric transection are crucial considerations. Previous studies have further recommended using a bougie size of >40 Fr, initiating gastric transection 5 to 6 cm from the pylorus, and avoiding the gastroesophageal junction.^[13,17]^ Furthermore, a narrow gastric tube and distal obstruction may increase the intraluminal pressure, further contributing to the risk of leakage.^[18–20]^ These factors could significantly contribute to the development of sleeve gastrectomy leakage. In the present case, abdominal CT revealed dilation of the upper sleeve-shaped stomach, whereas the lower sleeve-shaped stomach was not distended, suggesting increased intraluminal pressure due to stenosis.

Management of LSG-related leaks depends on several factors, including the leak location, size; and diameter of the leak orifice, and condition of the surrounding tissue. In addition, the patient’s nutritional status, presence of stenosis, and signs of peritonitis are crucial considerations when determining an appropriate treatment approach.^[1–4]^ Some reviews have indicated that staple line leaks typically resolve with conservative treatment within approximately 6.9 to 8.8 weeks.^[1,4,21]^ Several conservative endoscopic approaches have been described as initial treatments, including stent placement,^[22,23]^ EVT,^[1,24,25]^ OTSC,^[10,26]^ endoscopic internal drainage,^[27]^ fibrin glue,^[28]^ endoscopic septotomy,^[29,30]^ endoscopic balloon dilatation,^[31]^ and percutaneous transesophageal gastro-tubing.^[21]^

EVT is a recognized conservative endoscopic approach for managing SLL after SLG, offering benefits such as effective drainage, increased local blood flow, and stimulation of granulation tissue formation.^[24,25]^ Some studies have reported EVT to have a success rate of 89%^[24]^ and 87.5%.^[25]^ Additionally, the use of EVT for treating post-LSG gastric leaks has been associated with recovery times ranging from approximately 48.2 to 72.5 days.^[24,32]^ In our case, we also applied as a first-line conservative treatment for 94 days; however, it did not ultimately achieve successful resolution of the leakage.

The OTSC is another endoscopic technique used to treat SLL. The effectiveness of OTSC depends on the conditions of the tissue surrounding the leak.^[1,10,26]^ Keren et al^[10]^ reported a high success rate, achieving resolution in 21 of 26 patients (80.7%), while another study reported a success rate of 86.3%^[26]^ for leakage after sleeve gastrectomy. However, in some cases of chronic gastrointestinal fistula, the success rate of OTSC is approximately 40%, owing to the fibrotic edges of the lesion.^[12,26,33]^ In our case, we also used an OTSC, however it failed because of severe fibrosis near the fistula site and location of the fistula. Additionally, we summarized various endoscopic approaches for managing gastric leakage following sleeve gastrectomy in Table 1.^[10,21–31]^

**Table 1:** Summary of endoscopic treatment options and outcomes for managing gastric leakage after sleeve gastrectomy.

Author, year	Number of patients (N)	Treatment option	Success rate (%)	Conversion to salvage procedure	Post-procedure complications	Mortality rate (N)
Martínez et al^[22]^	488	Stent	85.8%	13.5% (n = 66) (reoperation)	18.65% (n = 91) (Stent migration)	n = 10
Rogalski et al^[23]^	344	Stent	92%	_	23% (n = 79) (Stent migration)	_
Leeds et al^[24]^	9	EVT	89%	11% (n = 1) (total gastrectomy)	22.2% (n = 2) (pancreatitis)	n = 1
Archid et al^[25]^	8	EVT	87.5%	_	12.5% (n = 1) (gastric bleeding)	_
Shoar et al^[26]^	73	OTSC	86.3%	_	19.2% (n = 14) (re-leakage, clip migration, stenosis, and tears)	_
Keren et al^[10]^	26	OTSC	80.7%	_	None	_
Siddique et al^[27]^	20	EID	85%	15% (n = 3) (gastric bypass [n = 2], fistula-jejunostomy [n = 1])	10% (n = 2) (gastrobronchial fistulas)	_
Assalia A et al^[28]^	24	Fibrin glue	95.8%	4.2% (n = 1) (OTSC)	_	_
Diaz et al^[29]^	5	Endoscopic septotomy	80%	20% (n = 1) (total gastrectomy)	None	None
Kim et al^[30]^	1	Endoscopic septotomy	100%	_	None	None
Campos et al^[31]^	1	EBD	100%	Combination with endoscopic septotomy for chronic leakage	None	None
Oshiro et al^[21]^	2	PTEG	100%	None	None	None

EBD = endoscopic band dilatation, EID = endoscopic internal drainage, EVT = endoscopic vacuum therapy, OTSC = over-the-scope-clips, PTEG = percutaneous transesophageal gastrotubing.

For a chronic leakage or fistula, endoscopic treatment is generally considered ineffective; thus, surgical intervention is a reliable option.^[1,9,34]^ Chronic leakage after an LSG may be influenced by factors inherent to the procedure. A high-pressure gastric tube caused by angular stenosis may adversely affect the healing process of the leak and potentially prolong its duration.^[11]^ Various treatment options have been proposed for chronic fistulae after LSG. The choice of surgical approach depends on the characteristics of the leak and quality of the surrounding tissue. If the leak site cannot be identified due to severe inflammation and adhesion, or if surrounding tissue is too fragile and fibrotic for anastomosis, TG with Roux-en-Y esophagojejunostomy may be an effective option for chronic fistulas.^[1,11,35]^

Roux-en-Y fistula-jejunostomy (RYFJ) is the most performed procedure for chronic SLL because it avoids the need for total or proximal gastrectomy. However, the conversion rate to more extensive surgical intervention, including open total gastrectomy, is estimated to be approximately 7.1% to 7.3%.^[11,36]^ In a literature review by Nedelcu et al,^[11]^ total gastrectomy for chronic fistula after sleeve gastrectomy demonstrated a lower rate (7.7%) of postoperative leak than other reconstructive surgeries, including RYFJ and Roux-en-Y gastric bypass, which reported leakage recurrence rates of leakage, 21.9% and 37.5%, respectively. Additionally, the mortality rates were 2.53% and 12.5% for RYFJ and Roux-en-Y gastric bypass, respectively.

Chronic fistulae typically require surgical intervention. This timeline aligns with the empirical knowledge of the fact that adhesion formation is the most severe at 2 weeks postoperatively and tends to decrease by approximately 3 months. The findings of animal studies support this clinical understanding. In one study, scratches were induced on the cecum and abdominal wall of mice, and adhesion formation was evaluated on postoperative days 7, 14, 21, and 28 after applying anti-adhesive agents. The results showed that the degree and strength of adhesions progressively increases until the 14th day, remained constant or increased slightly by the 21st day, and then began to decrease by the 28th day.^[37]^ These findings provide a scientific basis for the clinical approach of delaying surgical intervention for chronic fistulas until after 12 weeks; as this period allows for a natural reduction in adhesions, potentially improving the outcomes of surgical management.

In the present case, the patient’s condition was critical because of hemodynamic instability, and the initial CT findings revealed gastric perforation with dilation limited to the upper stomach. Thus, we hypothesized that perforation following sleeve gastrectomy and its failure to heal were likely due to elevated intragastric pressure. This remains a plausible explanation despite the absence of direct measurements of gastric pressure. Additionally, the initial exploratory examination revealed severe abdominal inflammation and adhesions, which prevented identification of the leakage site. Given the significant risks of morbidity and mortality associated with surgical intervention under these circumstances, we decided to not proceed with surgery at that stage. Consequently, EVT was selected as the first-line treatment for the leakage. However, this approach is ineffective at facilitating fistula healing. We therefore hypothesized that the EVT failure was attributed to the patient’s poor overall condition, elevated intragastric pressure, and persistent instability. After 97 days, a complete TG was performed. Although leakage persisted following this procedure, it resolved within 10 days. This outcome supports the hypothesis that elevated intragastric pressure is a key factor contributing to both the initial perforation and persistence of refractory fistulas.

## 4. Conclusion

Gastric leakage after LSG is one of the most challenging complications of MBS. Based on our experience, surgeons must carefully evaluate the patient’s overall condition and status of the leakage, including its location and size as well as the condition of the surrounding tissues. Conservative treatment may be a prudent first-line approach in cases of severe adhesion and poor patient condition. If conservative treatment fails to resolve a chronic fistula, TG may be considered as the definitive treatment option for refractory or chronic fistulas.

Our case suggests that increased intragastric pressure may play a significant role in contributing to sleeve gastrectomy leakage and persistence of refractory fistulas. These findings emphasizes the importance of addressing intragastric pressure to prevent and manages such complications. Clinically, if increased intragastric pressure is determined to be a key cause of chronic fistulas, the completion TG could serve as a useful treatment option.

## Acknowledgments

We wish to sincerely thank the patient for allowing her case to be presented.

## Author contributions

**Conceptualization:** Dae Hoon Kim.

**Data curation:** Gerelt-Od Khenmedekh, Dae Hoon Kim.

**Supervision:** Dae Hoon Kim.

**Writing – original draft:** Gerelt-Od Khenmedekh, Dae Hoon Kim.

**Writing – review & editing:** Dae Hoon Kim.

## References

[1] Oshiro T Wakamatsu K Nabekura T. Treatments for staple line leakage after laparoscopic sleeve gastrectomy. J Clin Med. 2023;12:3495. 37240601 10.3390/jcm12103495 PMC10219311

[2] Kheirvari M Dadkhah Nikroo N Jaafarinejad H. The advantages and disadvantages of sleeve gastrectomy; clinical laboratory to bedside review. Heliyon . 2020;6:e03496. 32154399 10.1016/j.heliyon.2020.e03496 PMC7052082

[3] Nimeri A Ibrahim M Maasher A Al Hadad M. Management algorithm for leaks following laparoscopic sleeve gastrectomy. Obes Surg. 2016;26:21–5. 26071239 10.1007/s11695-015-1751-2

[4] Bashah M Khidir N El-Matbouly M. Management of leak after sleeve gastrectomy: outcomes of 73 cases, treatment algorithm and predictors of resolution. Obes Surg. 2020;30:515–20. 31707571 10.1007/s11695-019-04203-w

[5] Benedix F Poranzke O Adolf D. Staple line leak after primary sleeve gastrectomy-risk factors and mid-term results: do patients still benefit from the weight loss procedure? Obes Surg. 2017;27:1780–8. 28078641 10.1007/s11695-017-2543-7

[6] Yolsuriyanwong K Ingviya T Kongkamol C Marcotte E Chand B. Effects of intraoperative leak testing on postoperative leak-related outcomes after primary bariatric surgery: an analysis of the MBSAQIP database. Surg Obes Relat Dis. 2019;15:1530–40. 31474524 10.1016/j.soard.2019.06.008

[7] Kim J Azagury D Eisenberg D DeMaria E Campos GM; American Society for Metabolic and Bariatric Surgery Clinical Issues Committee. ASMBS position statement on prevention, detection, and treatment of gastrointestinal leak after gastric bypass and sleeve gastrectomy, including the roles of imaging, surgical exploration, and nonoperative management. Surg Obes Relat Dis. 2015;11:739–48. 26071849 10.1016/j.soard.2015.05.001

[8] Chouillard E Chahine E Schoucair N. Roux-En-Y Fistulo-Jejunostomy as a salvage procedure in patients with post-sleeve gastrectomy fistula. Surg Endosc. 2014;28:1954–60. 24566743 10.1007/s00464-014-3424-y

[9] Bruzzi M Douard R Voron T Berger A Zinzindohoue F Chevallier JM. Open total gastrectomy with Roux-en-Y reconstruction for a chronic fistula after sleeve gastrectomy. Surg Obes Relat Dis. 2016;12:1803–8. 27387695 10.1016/j.soard.2016.03.013

[10] Keren D Eyal O Sroka G. Over-the-scope clip (OTSC) system for sleeve gastrectomy leaks. Obes Surg. 2015;25:1358–63. 25511753 10.1007/s11695-014-1540-3

[11] Nedelcu M Danan M Noel P Gagner M Nedelcu A Carandina S. Surgical management for chronic leak following sleeve gastrectomy: review of literature. Surg Obes Relat Dis. 2019;15:1844–9. 31588005 10.1016/j.soard.2019.03.015

[12] Aurora AR Khaitan L Saber AA. Sleeve gastrectomy and the risk of leak: a systematic analysis of 4,888 patients. Surg Endosc. 2012;26:1509–15. 22179470 10.1007/s00464-011-2085-3

[13] Jain N Bhojwani R Mahawar K. Leaks after sleeve gastrectomy – a narrative review. J Bariatric Surg. 2012;1:2–9.

[14] Rebibo L Tricot M Dembinski J Dhahri A Brazier F Regimbeau JM. Gastric leak after sleeve gastrectomy: risk factors for poor evolution under conservative management. Surg Obes Relat Dis. 2021;17:947–55. 33640258 10.1016/j.soard.2021.01.023

[15] Nienhuijs SW Kaymak U Korsten E Buise MP. Influence of intraoperative hypotension on leaks after sleeve gastrectomy. Surg Obes Relat Dis. 2016;12:535–9. 26656668 10.1016/j.soard.2015.08.506

[16] Benedix F Benedix DD Knoll C. Are there risk factors that increase the rate of staple line leakage in patients undergoing primary sleeve gastrectomy for morbid obesity? Obes Surg. 2014;24:1610–6. 24748473 10.1007/s11695-014-1257-3

[17] Parikh M Issa R McCrillis A Saunders JK Ude-Welcome A Gagner M. Surgical strategies that may decrease leak after laparoscopic sleeve gastrectomy: a systematic review and meta-analysis of 9991 cases. Ann Surg. 2013;257:231–7. 23023201 10.1097/SLA.0b013e31826cc714

[18] Masood M Low DE Deal SB Kozarek RA. Endoscopic management of post-sleeve gastrectomy complications. J Clin Med. 2024;13:2011. 38610776 10.3390/jcm13072011 PMC11012813

[19] Boules M Chang J Haskins IN. Endoscopic management of post-bariatric surgery complications. World J Gastrointest Endosc. 2016;8:591–9. 27668069 10.4253/wjge.v8.i17.591 PMC5027029

[20] Casella G Soricelli E Rizzello M. Nonsurgical treatment of staple line leaks after laparoscopic sleeve gastrectomy. Obes Surg. 2009;19:821–6. 19381737 10.1007/s11695-009-9840-8

[21] Oshiro T Saiki A Suzuki J. Percutaneous transesophageal gastro-tubing for management of gastric leakage after sleeve gastrectomy. Obes Surg. 2014;24:1576–80. 24917053 10.1007/s11695-014-1322-y

[22] Martinez Hernandez A Beltran Herrera H Martinez Garcia V. Stent management of leaks after bariatric surgery: a systematic review and meta-analysis. Obes Surg. 2022;32:1034–48. 35132569 10.1007/s11695-022-05890-8

[23] Rogalski P Swidnicka-Siergiejko A Wasielica-Berger J. Endoscopic management of leaks and fistulas after bariatric surgery: a systematic review and meta-analysis. Surg Endosc. 2021;35:1067–87. 32107632 10.1007/s00464-020-07471-1 PMC7886733

[24] Leeds SG Burdick JS. Management of gastric leaks after sleeve gastrectomy with endoluminal vacuum (E-Vac) therapy. Surg Obes Relat Dis. 2016;12:1278–85. 27178614 10.1016/j.soard.2016.01.017

[25] Archid R Wichmann D Klingert W. Endoscopic vacuum therapy for staple line leaks after sleeve gastrectomy. Obes Surg. 2020;30:1310–5. 31792702 10.1007/s11695-019-04269-6

[26] Shoar S Poliakin L Khorgami Z. Efficacy and safety of the over-the-scope clip (OTSC) system in the management of leak and fistula after laparoscopic sleeve gastrectomy: a systematic review. Obes Surg. 2017;27:2410–8. 28353180 10.1007/s11695-017-2651-4

[27] Siddique I Alazmi W Al-Sabah SK. Endoscopic internal drainage by double pigtail stents in the management of laparoscopic sleeve gastrectomy leaks. Surg Obes Relat Dis. 2020;16:831–8. 32389513 10.1016/j.soard.2020.03.028

[28] Assalia A Ilivitzki A Ofer A. Management of gastric fistula complicating laparoscopic sleeve gastrectomy with biological glue in a combined percutaneous and endoscopic approach. Surg Obes Relat Dis. 2018;14:1093–8. 29895427 10.1016/j.soard.2018.04.009

[29] Diaz R Welsh LK Perez JE. Endoscopic septotomy as a treatment for leaks after sleeve gastrectomy: meeting presentations: digestive disease week 2019. Endosc Int Open. 2020;8:E70–5. 31921987 10.1055/a-1027-6888 PMC6949161

[30] Kim KH Jung K Kim YH Seo KW. Endoscopic septotomy as a treatment for chronic leak after laparoscopic sleeve gastrectomy. J Metab Bariatr Surg. 2021;10:42–5. 36687751 10.17476/jmbs.2021.10.1.42 PMC9847649

[31] Campos JM Ferreira FC Teixeira AF. Septotomy and balloon dilation to treat chronic leak after sleeve gastrectomy: technical principles. Obes Surg. 2016;26:1992–3. 27299918 10.1007/s11695-016-2256-3

[32] Parmer M Wang YHW Hersh EH Zhang L Chin E Nguyen SQ. Management of staple line leaks after laparoscopic sleeve gastrectomy. JSLS. 2022;26:e2022.00029. 10.4293/JSLS.2022.00029 PMC9439287 36071996

[33] Lee HL Cho JY Cho JH. Efficacy of the over-the-scope clip system for treatment of gastrointestinal fistulas, leaks, and perforations: a Korean multi-center study. Clin Endosc. 2018;51:61–5. 28847073 10.5946/ce.2017.027 PMC5806921

[34] Rosenthal RJ Arvidsson D Baker RS. International sleeve gastrectomy expert panel consensus statement: best practice guidelines based on experience of >12,000 cases. Surg Obes Relat Dis. 2012;8:8–19. 22248433 10.1016/j.soard.2011.10.019

[35] Lainas P Schoucair N Dammaro C Dagher I. Salvage procedures for chronic gastric leaks after sleeve gastrectomy: the role of laparoscopic Roux-en-Y fistulo-jejunostomy. Ann Transl Med. 2019;7(Suppl 3):S119. 31576326 10.21037/atm.2019.05.40 PMC6685896

[36] Lainas P Triantafyllou E Ben Amor V. Laparoscopic Roux-en-Y fistulojejunostomy as a salvage procedure in patients with chronic gastric leak after sleeve gastrectomy. Surg Obes Relat Dis. 2023;19:585–92. 36658084 10.1016/j.soard.2022.12.017

[37] Lee YM Lee YW Evaluation on effectiveness for preventing post surgical adhesion of sodium hyaluronate/sodium carboxymethyl cellulose (HA/CMC) membrane in rat cecum/peritonium mode. Membr J . 2005;15:213–23.

